# A Modified Method for Cerebrospinal Fluid Collection in Anesthetized Rat and Evaluation of the Efficacy 

**Published:** 2013

**Authors:** Amin Zarghami, Farid Alinezhad, Sareh Pandamooz, Mohammad Naji, Mohsen Pourghasem

**Affiliations:** 1 *Student Research Committee, Babol University of Medical Sciences, Babol, Iran.*; 2 *Cellular and Molecular Biology Research Center (CMBRC), Babol University of Medical Sciences, Babol, Iran.*; 3 *Department of Anatomy, School of Medicine, Tehran University of Medical Sciences, Tehran, Iran.*; 4 *Department of Anatomy and Embryology, Babol University of Medical Sciences, Babol, Iran.*

The role of the cerebrospinal fluid (CSF) as a transport medium for biologically and behaviorally active hormones, drugs, and other such molecules has become increasingly evident over the past decade. It is a good sample for the reflection of biological changes inside the brain (1). Several studies have utilized different methods for collecting the CSF from the anesthetized or non-anesthetized rats, such as: involving implantation of cannula catheters into cisterna magna or to drill a hole in the calvarium and insert a tube either subdurally or extradurally along the occipital bone; but these approaches could not be sufferable and cause serious complications to brain parenchyma. Another method is direct lumbar puncture but the problem is, its biochemical composition may not totally reflect the neurological function for studies conducted in cranial region. On the other hand, blood contamination of the CSF is common for all of these established techniques (2-3). It is well known that cisterna magna have the largest volume of CSF in the rat CNS. In the present experimental study, we evaluate a simplified technique for efficient collection of CSF from cisterna magna of the adult rats.

**Figure 1:**
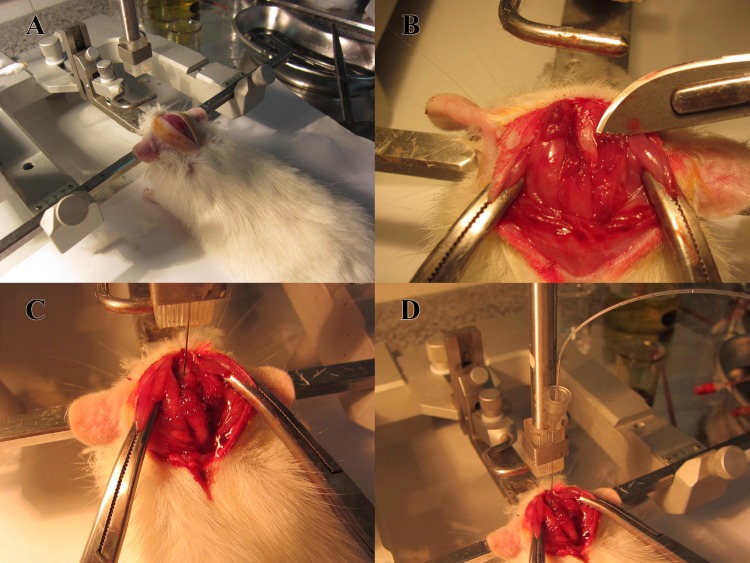
A: Rat was placed in a stereotaxic head holder. The head was ﬂexed downward at approximately 90◦. A midline scalp incision was made and the cervico-spinal muscle was reflected. B: The atlanto-ocipital membrane exposed. C: The atlanto-occipital membrane punctured by the fire-polished 1 ml syringe connected to the 27G dental needle. D: Using a special stereotaxic guide to hold the syringe

Twenty three male Wistar rats with the body weight range of 200-300 g were enrolled in the study. Rats were anesthetized by ketamine(50 mg/kg) and xylasine (10mg/kg) intraperitoneally. Specially constructed ear bars were placed in the external auditory meatus and the rats were placed in a stereotaxic frame (Stoelting Inc, USA) (Fig. 1). The head was ﬂexed downward at approximately 90◦, a depressible surface with the appearance of a rhomb between occipital protuberances and the spine of the atlas becomes palpable. A midline scalp incision was made and the cervico-spinal muscle was reflected and the atlanto-occipital membrane exposed. Using a special stereotaxic guide to hold the syringe, the point was carefully advanced under direct vision. The atlanto-occipital membrane punctured by the fire-polished 1 ml syringe connected to the 27G dental needle. Then by a gentle aspiration, the non-contaminated CSF was drawn into the syringe. We perform this method by working with two people, one for needle insertion into cisterna magna by the stereotax and control the system for any color change or extra movements, and another for aspirating the syringe. Seventeen rats were obtained for CSF collection. The successful index was the collection of clear and colorless CSF. In thirteen rats, we could succ-essfully aspirate the CSF. The total volume was 1200 micro liters (varies from 80 to150 µlit per animal). The success rate was 76.4%. Failure etiologies were mainly related to dry aspiration, blood contaminated CSF and obstruction of the needle.

This simple technique represented an efficient method for experiments in which non-contaminated CSF are required. The required materials are cheap and easy to be obtained. The technique could be easily done by anyone who is familiar with stereotaxic surgery.

Sincerely.
